# Buccal mucosal graft urethroplasty for radiation-induced urethral strictures: an evaluation using the extended Urethral Stricture Surgery Patient-Reported Outcome Measure (USS PROM)

**DOI:** 10.1007/s00345-020-03102-5

**Published:** 2020-02-18

**Authors:** Malte W. Vetterlein, Luis A. Kluth, Valentin Zumstein, Christian P. Meyer, Tim A. Ludwig, Armin Soave, Silke Riechardt, Oliver Engel, Roland Dahlem, Margit Fisch, Clemens M. Rosenbaum, for the Trauma and Reconstructive Urology Working Party of the European Association of Urology Young Academic Urologists

**Affiliations:** 1 Department of Urology, University Medical Center Hamburg-Eppendorf, Martinistr. 52, 20246 Hamburg, Germany; 2 Department of Urology, University Medical Center Frankfurt, Frankfurt (Main), Germany; 3 Department of Urology, Cantonal Medical Center St. Gallen, St. Gallen, Switzerland; 4 Department of Urology, Asklepios Medical Center Barmbek, Hamburg, Germany

**Keywords:** Lower urinary tract symptoms, Patient satisfaction, Quality of life, Radiotherapy, Urinary incontinence

## Abstract

**Objectives:**

To evaluate objective treatment success and subjective patient-reported outcomes in patients with radiation-induced urethral strictures undergoing single-stage urethroplasty.

**Patients and methods:**

Monocentric study of patients who underwent single-stage ventral onlay buccal mucosal graft urethroplasty for a radiation-induced stricture between January 2009 and December 2016. Patients were characterized by descriptive analyses. Kaplan–Meier estimates were employed to plot recurrence-free survival. Recurrence was defined as any subsequent urethral instrumentation (dilation, urethrotomy, urethroplasty). Patient-reported functional outcomes were evaluated using the validated German extension of the Urethral Stricture Surgery Patient-Reported Outcome Measure (USS PROM).

**Results:**

Overall, 47 patients were available for final analyses. Median age was 70 (IQR 65–74). Except for two, all patients had undergone pelvic radiation therapy for prostate cancer. Predominant modality was external beam radiation therapy in 70% of patients. Stricture recurrence rate was 33% at a median follow-up of 44 months (IQR 28–68). In 37 patients with available USS PROM data, mean six-item LUTS score was 7.2 (SD 4.3). Mean ICIQ sum score was 9.8 (SD 5.4). Overall, 53% of patients reported daily leaking and of all, 26% patients underwent subsequent artificial urinary sphincter implantation. Mean IIEF-EF score was 4.4 (SD 7.1), indicating severe erectile dysfunction. In 38 patients with data regarding the generic health status and treatment satisfaction, mean EQ-5D index score and EQ VAS score was 0.91 (SD 0.15) and 65 (SD 21), respectively. Overall, 71% of patients were satisfied with the outcome.

**Conclusion:**

The success rate and functional outcome after BMGU for radiation-induced strictures were reasonable. However, compared to existing long-term data on non-irradiated patients, the outcome is impaired and patients should be counseled accordingly.

## Introduction

Despite the increasing uptake of active surveillance, interventional therapies such as radiation therapy (RT) and radical prostatectomy represent a cornerstone in the treatment of localized prostate cancer (PCa) [1]. Notwithstanding the proven efficacy of ionizing radiation and given its widespread use, 5-year lower urinary tract toxicities can be as high as 13% [2], of which urethral stricture is the predominant subtype [3]. In a recent meta-analysis of roughly 16,000 patients, the pooled estimate of urethral stricture prevalence following RT for PCa was 2.2% at a median follow-up of 4 years [4]. Of note, stricture prevalence was higher in case of combination therapy (brachytherapy + external beam RT) [4] and these modality-dependent trends were corroborated in a SEER-Medicare linked cohort with 10-year propensity-weighted cumulative incidences of 9.6%, 12%, and 19% after brachytherapy, external beam RT, and combination therapy, respectively [3].

While the available evidence on urethroplasty for radiation-induced strictures is very limited, reports are congruent regarding a significantly shorter recurrence-free survival compared to strictures of non-radiogenic etiology [5–8]. Such complexity is mainly promoted by prostatic radionecrosis [9], proximity to the urinary sphincter due to the bulbomembranous location in the majority of cases [4], and impaired tissue vascularity [10]. The controversy regarding the optimal surgical technique to treat radiation-induced urethral strictures is driven by two main considerations: some authors prefer excision and primary anastomosis given the presumed poor tissue vascularity [7, 9] whereas others prefer grafts or flaps due to stricture lengths of mostly ≥ 2 cm [5, 11]. In the absence of level I evidence to prove the superiority of one technique over the other, all of those reports suffer from a lack of urethral stricture-specific validated patient-reported outcome measurements (PROMs) [12]. Particularly in patients with a history of RT, it is highly relevant to assess urinary incontinence (UI), erectile dysfunction (ED), and quality of life in a standardized fashion, as many patients are rendered incontinent after urethroplasty and may present with ED due to extensive radiation fibrosis.

Against this backdrop, the aim of our study was to evaluate the success rate in a homogeneous cohort of patients with radiation-induced urethral strictures undergoing single-stage buccal mucosal graft urethroplasty (BMGU) using the extended Urethral Stricture Surgery (USS) PROM.

## Patients and methods

### Study population

This was an institutional review board-approved monocentric retrospective analysis of patients who underwent single-stage BMGU for radiation-induced urethral stricture between January 1, 2009 and December 31, 2016. For the purpose of this study, we only selected patients who had undergone previous RT or ablative therapy of the pelvis and who subsequently presented with a radiation-induced bulbomembranous urethral stricture at our department.

### Evaluation, surgical procedure, and perioperative management

The standardized institutional perioperative workflow has been previously described in detail [13, 14]. After preoperative stricture evaluation via a combined retrograde urethrography and voiding cystourethrography, single-stage ventral onlay BMGU was performed in all patients according to a previously described technique [15]. Transurethral catheterization was performed for 10 days and cystostomy for 21 days until the patient revisited the outpatient clinic to perform a standardized radiographic and functional voiding trial [13].

### Follow-up, the definition of stricture recurrence, and patient-reported outcome measurement

We individually reviewed digital charts for readmissions and revisits to our institution. Patients were contacted personally for follow-up via phone or mail given that many patients were nationwide referrals and the individual follow-up was performed by the respective local urologist, and stricture recurrence was defined as the symptomatic need for any instrumentation (dilation, endoscopic or open reconstructive surgery) [13]. For patient-reported outcome analysis, we used the psychometrically validated and extended German translation [16] of the previously developed and validated USS PROM tool by Jackson and his colleagues [17]. The USS PROM is a composite instrument incorporating a LUTS domain (ICSmale short form voiding score, a single item from the ICSmale short form incontinence score, and a single item from the ICSQoL questionnaire) [18], Peeling’s voiding picture [19], a generic health status domain (EQ-5D-3L of the EuroQol group) [20], and a treatment satisfaction question [17]. Our 2013 extension of the USS PROM additionally comprises validated questionnaires regarding erectile function (IIEF erectile function domain) [21] and UI (ICIQ UI short form) [22].

### Covariates

We collected baseline characteristics (age, ASA™ score, body mass index, and select comorbidities) as well as detailed information on previous treatments (RT indication and modality, and whether PCa surgery had been performed beforehand). We furthermore evaluated the history of previous interventions for urethral stricture (urethrotomy and urethroplasty). Surgical characteristics comprised operative time, graft length, surgical volume, and length of hospital stay.

### Statistical analyses

Our statistical analyses consisted of several steps. First, baseline, treatment, and surgical characteristics were summarized by descriptive analyses. The distribution of categorical variables was reported using frequencies and proportions, whereas continuous variables were reported using means and standard deviations or medians and interquartile ranges, depending on whether variables were normally or non-normally distributed, which was tested by the Shapiro–Wilk test.

Second, median follow-up time in censored patients was calculated using reverse Kaplan–Meier estimates and a Kaplan–Meier curve was plotted to depict stricture-recurrence free survival.

Third, PROM scoring was performed as previously described in detail [17]. Shortly, we performed descriptive analyses of functional outcomes as represented by the USS PROM [17] and our extension [16]. For each domain (LUTS, UI, and ED), frequencies and proportions of different responses were reported. In addition, the total mean six-item LUTS score, ICIQ sum score, and IIEF-EF score were calculated. The EQ visual analogue scale (EQ VAS) was used to elicit respondents’ global health rating on a scale from 0 to 100 and health profiles were converted to EQ-5D index values using German population preference weights derived from a time trade-off survey [23].

All statistical analyses were performed using Stata^®^ (StataCorp. 2015. Stata Statistical Software: Release 14. College Station, TX: StataCorp LP). The reported *P* values were two-sided and values < 0.05 were considered statistically significant.

## Results

### Study population

Overall, 47 patients were available for final analyses. Median age was 70 (IQR 65–74) and 45 patients (96%) had an ASA™ score of 2 or 3, indicating mild or severe systemic disease, respectively. Of all patients, 17 (36%), 10 (21%), and 32 (68%) suffered from coronary heart disease, diabetes mellitus, and hypertension, respectively, and 22 patients (47%) were under antiplatelet or anticoagulant therapy. Except for two patients, who had been radiated for rectal or bladder cancer, all patients had undergone prior pelvic RT for PCa. The predominant RT modality was external beam RT in 33 patients (70%), followed by combined high dose rate brachytherapy plus external beam RT in eight patients (17%), and low dose rate brachytherapy in five patients (11%). One patient (2.1%) underwent high-intensity focused ultrasound. Overall, 24 patients (51%) had undergone radical prostatectomy and 29 (62%) had a history of at least one previous urethrotomy. Conversely, only two patients (4.2%) had a history of previous urethroplasty (Table 1). Median operative time was 73 min (IQR 58–93) and median graft length was 5 cm (IQR 4–5). In 31 cases (66%), the procedure was performed by two experienced senior high-volume surgeons (i.e. ≥ 100 BMGUs over the study period) and the remaining 16 patients (34%) were operated by intermediate volume surgeons of our department (i.e. < 100 BMGUs). Median length of stay was 6 days (IQR 6–7).

**Table 1:** Baseline, treatment, and surgical characteristics of patients who underwent single-stage buccal mucosal graft urethroplasty for radiation-induced bulbomembranous urethral stricture

Patient number; *N* (%)	47 (100)
Age at surgery (years); median (IQR)	70 (65–74)
ASA™ score; *N* (%)	
1	2 (4.3)
2	22 (47)
3	23 (49)
4	0 (0)
Body mass index; median (IQR)	27 (26–30)
Coronary heart disease; *N* (%)	17 (36)
Diabetes mellitus; *N* (%)	10 (21)
Hypertension; *N* (%)	32 (68)
Smoking status; *N* (%)	
Never	19 (40)
Ever	28 (60)
Anticoagulants/antiplatelets; *N* (%)	
None	25 (53)
Aspirin	15 (32)
Clopidogrel	1 (2.1)
Oral anticoagulants*	6 (13)
RT indication; *N* (%)	
Prostate cancer	45 (96)
Rectal cancer	1 (2.1)
Bladder cancer	1 (2.1)
RT modality; *N* (%)	
LDR brachytherapy	5 (11)
External beam RT	33 (70)
HDR brachytherapy + external beam RT	8 (17)
High-intensity focused ultrasound	1 (2.1)
Prostate cancer surgery (*N* = 45); *N* (%)	
None	12 (26)
Radical prostatectomy	24 (51)
TURP^†^	10 (21)
Retropubic adenomectomy	1 (2.1)
History of direct vision internal urethrotomy; *N* (%)	
None	18 (38)
1	12 (26)
2–5	15 (32)
≥ 6	2 (4.3)
History of urethroplasty; *N* (%)	
None	45 (96)
1	1 (2.1)
≥ 2	1 (2.1)

Proportions may not add up to 100%, as they are rounded

*ASA™* American Society of Anesthesiologists™, *HDR* high dose rate, *IQR* interquartile range, *LDR* low dose rate, *RT* radiation therapy, *TURP* transurethral resection of the prostate

*Patients with oral anticoagulants were switched to a low-molecular-weight heparin preoperatively

^†^One patient underwent TURP and subsequent high-intensity focused ultrasound

### Follow-up and stricture recurrence-free survival

**Fig. 1:**
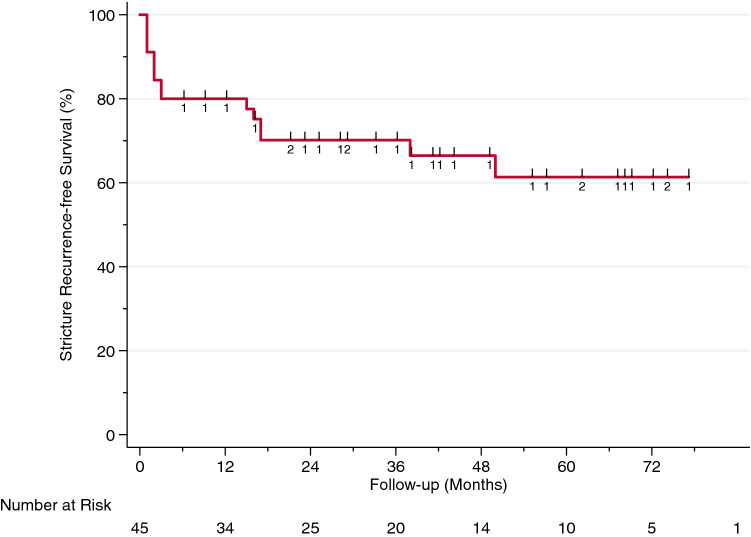
Kaplan–Meier analysis of stricture recurrence-free survival in 45 men who underwent single-stage buccal mucosal graft urethroplasty for radiation-induced bulbomembranous urethral stricture

Overall, two patients (4.2%) were lost to follow-up and were excluded prior to survival analyses. Median follow-up in all patients was 44 months (IQR 28–68) and 15 of 45 (33%) had stricture recurrence. Median time to recurrence was 3 months (IQR 1–17). The Kaplan–Meier curve and the corresponding risk table are depicted in Fig. 1.

### Patient-reported outcome measures (extended USS PROM)

Overall, the complete extended postoperative USS PROM was available in 34 of 47 patients, which translates into a response rate of 72%.

- a. Lower urinary tract symptoms (LUTS)
- The total six-item mean LUTS score (0–24) was 7.2 (SD 4.3). In the different LUTS items, 28 (83%), 25 (74%), 18 (53%), 29 (86%), 22 (65%), and ten patients (30%) reported no or just occasional problems regarding voiding delay, strength of urinary stream, the need to strain to continue urinating, stop-and-start issues, complete bladder emptying, and urinary dripping, respectively. Overall, ten patients (30%) reported an interference of urinary symptoms with their life (Table 2). Mean Peeling’s stream picture score was 2.7 (SD 1.1).

**Table 2:** Frequencies and proportions of patient responses to summative questions of the lower urinary tracts symptoms domain within the validated USS PROM tool in patients who underwent single-stage buccal mucosal graft urethroplasty for radiation-induced bulbomembranous urethral stricture

Item	Frequencies (proportions, %)
Q1: Is there a delay before you start to urinate?	
Never	21 (62)
Occasionally	7 (21)
Sometimes	1 (2.9)
Most of the time	4 (12)
All the time	1 (2.9)
Q2: Would you say that the strength of your urinary stream is…	
Normal	18 (53)
Occasionally reduced	7 (21)
Sometimes reduced	5 (15)
Reduced most of the time	3 (8.8)
Reduced all of the time	1 (2.9)
Q3: Do you have to strain to continue urinating?	
Never	13 (38)
Occasionally	5 (15)
Sometimes	4 (12)
Most of the time	6 (18)
All the time	6 (18)
Q4: Do you stop and start more than once while you urinate?	
Never	24 (71)
Occasionally	5 (15)
Sometimes	2 (5.9)
Most of the time	1 (2.9)
All the time	2 (5.9)
Q5: How often do you feel your bladder has not emptied properly after you have urinated?	
Never	13 (38)
Occasionally	9 (27)
Sometimes	8 (24)
Most of the time	3 (8.8)
All the time	1 (2.9)
Q6: How often have you had a slight wetting of your pants a few minutes after you had finished urinating and had dressed yourself?	
Never	4 (12)
Occasionally	6 (18)
Sometimes	9 (27)
Most of the time	7 (21)
All the time	8 (24)
Six-item LUTS sum score; mean (SD)	7.2 (4.3)
Q7: Overall, how much do your urinary symptoms interfere with your life?	
Not at all	8 (24)
A little	16 (47)
Somewhat	7 (21)
A lot	3 (8.8)
Q8: Peeling’s voiding picture (*N* = 21)	
1	4 (19)
2	5 (24)
3	6 (29)
4	6 (29)

Proportions may not add up to 100%, as they are rounded

*BMGU* buccal mucosal graft urethroplasty, *LUTS* lower urinary tract symptoms, *SD* standard deviation, *USS PROM* Urethral Stricture Surgery Patient-reported Outcome Measure
- b. Urinary incontinence
- The total mean ICIQ sum score (0–21) was 9.8 (SD 5.4), corresponding to ‘moderate’ incontinence [24]. Overall, 18 (53%) and 11 patients (32%) reported to leak at least several times a day and at least a moderate amount of urine, respectively. There was no patient to report complete continence and there were three patients (8.8%) who reported a permanent incontinence (Table 3). According to the ICIQ-UI short form severity categories as defined by Klovning et al. [24], 48% of patients with previous prostatectomy and 20% of patients with previous TURP were severely (or very severely) incontinent (*P* = 0.4). Consequently, 12 of all 47 patients (26%) underwent artificial urinary sphincter (AMS 800™) implantation at a median interval of 5.8 months (IQR 3.7–14) after BMGU.

**Table 3:** Frequencies and proportions of patient responses to summative questions of the urinary incontinence domain within the validated USS PROM tool in patients who underwent single-stage buccal mucosal graft urethroplasty for radiation-induced bulbomembranous urethral stricture

Item	Frequencies (proportions, %)
Q1: How often do you leak urine?	
Never	4 (12)
About once a week or less often	6 (18)
Two or three times a week	2 (5.9)
About once a day	4 (12)
Several times a day	12 (35)
All the time	6 (18)
Q2: How much urine do you usually leak (whether you wear protection or not)?	
None	3 (8.8)
A small amount	20 (59)
A moderate amount	6 (18)
A large amount	5 (15)
Q3: Overall, how much does leaking urine interfere with your everyday life? Please ring a number between 0 (not at all) and 10 (a great deal)	
0	6 (18)
1	0 (0)
2	7 (21)
3	2 (5.9)
4	3 (8.8)
5	4 (12)
6	4 (12)
7	4 (12)
8	2 (5.9)
9	1 (2.9)
10	1 (2.9)
ICIQ sum score; mean (SD)	9.8 (5.4)
Q4: When does urine leak? (Please tick all that apply to you)	
Never—urine does not leak	0 (0)
Leaks before you can get to the toilet	6 (18)
Leaks when you cough or sneeze	7 (21)
Leaks when you are asleep	5 (15)
Leaks when you are physically active/exercising	10 (29)
Leaks when you have finished urinating and are dressed	2 (5.9)
Leaks for no obvious reason	8 (24)
Leaks all the time	3 (8.8)

Proportions may not add up to 100%, as they are rounded

*SD* standard deviation, *USS PROM* Urethral Stricture Surgery Patient-reported Outcome Measure
- c. Erectile function
- The mean IIEF-EF score (1 to 30) was 4.4 (SD 7.1) and at least 20 (59%), 24 (71%), and 30 patients (88%) reported no sexual activity, did not attempt intercourse and rated their confidence very low to get and keep an erection, respectively (Table 4).

**Table 4:** Frequencies and proportions of patient responses to summative questions of the erectile function domain (IIEF-EF) within the validated extension of the USS PROM tool in patients who underwent single-stage buccal mucosal graft urethroplasty for radiation-induced bulbomembranous urethral stricture

Q1: How often were you able to get an erection during sexual activity?	
No sexual activity	20 (59)
Almost never/never	9 (27)
A few times (much less than half the time)	3 (8.8)
Sometimes (about half the time)	0 (0)
Most times (much more than half the time)	0 (0)
Almost always/always	2 (5.9)
Q2: When you had erections with sexual stimulation, how often were your erections hard enough for penetration?	
No sexual activity	23 (68)
Almost never/never	7 (21)
A few times (much less than half the time)	2 (5.9)
Sometimes (about half the time)	0 (0)
Most times (much more than half the time)	0 (0)
Almost always/always	2 (5.9)
Q3: When you attempted sexual intercourse, how often were you able to penetrate (enter) your partner)	
Did not attempt intercourse	23 (68)
Almost never/never	7 (21)
A few times (much less than half the time)	2 (5.9)
Sometimes (about half the time)	0 (0)
Most times (much more than half the time)	0 (0)
Almost always/always	2 (5.9)
Q4: During sexual intercourse, how often were you able to maintain your erection after you had penetrated (entered) your partner?	
Did not attempt intercourse	24 (71)
Almost never/never	6 (18)
A few times (much less than half the time)	2 (5.9)
Sometimes (about half the time)	0 (0)
Most times (much more than half the time)	0 (0)
Almost always/always	2 (5.9)
Q5: During sexual intercourse, how difficult was it to maintain your erection to completion of intercourse?	
Did not attempt intercourse	27 (79)
Extremely difficult	3 (8.8)
Very difficult	0 (0)
Difficult	2 (5.9)
Slightly difficult	0 (0)
Not difficult	2 (5.9)
Q15: How do you rate your confidence that you could get and keep an erection?	
Very low	30 (88)
Low	2 (5.9)
Moderate	0 (0)
High	1 (2.9)
Very high	1 (2.9)
IIEF-EF score; mean (SD)	4.4 (7.1)

Proportions may not add up to 100%, as they are rounded

**Fig. 2:**
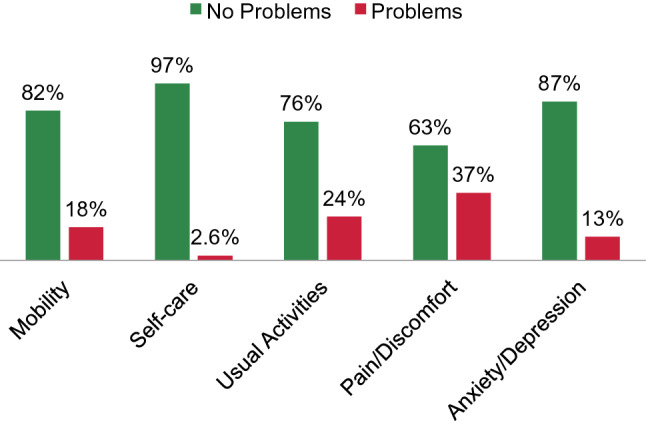
Proportions of patient-reported problems vs. no problems in the different EQ-5D generic health profiles in 38 men after single-stage buccal mucosal graft urethroplasty for radiation-induced bulbomembranous urethral stricture

*IIEF-EF* erectile function domain of the International Index of Erectile Function, *SD* standard deviation, *USS PROM* Urethral Stricture Surgery Patient-reported Outcome Measure
- d. Generic health status and treatment satisfaction
- Responses for the different generic health profiles and treatment satisfaction were available in 38 patients (79%). Eighteen men (47%) described themselves as being in full health in the EQ-5D descriptive system. The distributions of health profile responses are depicted in Fig. 2 and the Appendix Table. EQ-5D index scores showed a mean of 0.91 (SD 0.15) and mean EQ VAS score was 65 (SD 21). Furthermore, 27 patients (71%) were satisfied with the surgical outcome.

## Discussion

Our results confirm that the surgical management of radiation-induced strictures is more challenging compared to stricture repair for other reasons. With an overall recurrence rate of 33%, treatment success was significantly lower compared to BMGU in bulbar strictures of non-radiogenic etiology [13–15]. Radiation-induced urethral stricture following RT for PCa represents one of the most common late grade 2–3 urinary adverse events (UAEs) [3]. Inflammation and the associated tissue damage are considered an effect of oxidative stress as a result of ionizing radiation to actively divide cells [25, 26]. Hence, urethral strictures develop secondary to chronic fibrosis and progressive endarteritis in this context. Despite a lack of evidence regarding histopathological evaluation of radiation-induced strictures, a compromised vascular supply and poor wound healing may explain inferior outcomes.

Our findings are in line with previous data on BMGU in radiation-induced strictures. Whereas small case series by Meeks et al. [8] and Glass et al. [6] reported success in 50% and 80%, respectively, we observed an overall success rate of 71% in 38 BMGU patients in an earlier evaluation from our institution [5]. In comparison, published success rates after excision and primary anastomosis resulted in slightly better outcomes with 70–95% treatment success [6–8]. However, median graft length in our current series was 5 cm, implying a stricture length too long to be eligible for sufficient mobilization and primary anastomosis with promising results.

Besides the clinician-driven objective indicators of success such as recurrence-free survival, recent initiatives have focused on health-related quality of life measures and particularly, patient-centered outcomes [12]. To the best of our knowledge, no validated procedure-specific PROM tool has been used in patients undergoing urethroplasty for radiation-induced urethral strictures so far. In the current study, stricture-recurrence as objective outcome directly translated into worse patient-reported outcomes, as the mean six-item LUTS sum score was 7.2 (Table 2), which is higher compared to postoperative scores in patients undergoing BMGU for strictures of predominantly non-radiogenic etiology [16, 17, 27].

Besides stricture recurrence, UI represents one of the major UAEs after urethroplasty for radiation-induced strictures with de-novo UI rates ranging between 7 and 50% [5–8, 28]. While these cited reports mostly rely on functional outcomes after excision and primary anastomosis, Ahyai et al. reported a postoperative de-novo UI and overall UI in 11% and 42% of patients, respectively [5]. In our current study, mean postoperative ICIQ sum score was 9.8, which is higher compared to a mean of 3.4 in a predominantly non-radiogenic stricture reference cohort [16]. Furthermore, more than 50% of patients reported daily leaking and thus, our data corroborate the higher risk of UI following BMGU for radiation-induced strictures. Overall, one-third of patients with available PROMs reported a moderate or large amount of urine leak, which is highlighted by subsequent artificial urinary sphincter implantation in one-fourth of all patients. Intriguingly, severe or very severe UI was reported more often following radical prostatectomy (with adjuvant or salvage RT) compared to primary RT to the prostate, although this was not statistically significant due to the relatively small sample size. Arguably, high UI rates are likely associated with the previous PCa treatment, as UI is a well-known late UAE after primary external beam RT of the pelvis and even more so after radical prostatectomy and adjuvant RT [3].

Additionally, urgency represents another bothering UAE following urethroplasty for radiation-induced strictures [6, 29]. Roughly one in five patients reported urgency issues in the current series. Whether such problems are related to the pre-existing outlet obstruction or to radiogenic damage of the bladder itself remains uncertain. However, high rates of urgency and urge UI have been described for anterior urethroplasty of non-radiogenic etiology, suggesting a non-negligible intrinsic effect of the urethroplasty itself [30].

In the context of anterior urethroplasty for non-radiogenic strictures, ED is rare and mostly temporary [31]. Conversely, the evidence on ED in patients with radiation-induced strictures is controversial. Some studies reported a severe deterioration of erectile function following urethroplasty [29], others observed only a marginal change [5, 8]. In our series, we found that the majority reported no sexual activity at all (mean IIEF-EF score: 4.4). ED rates after RT are as high as 70% [32] and thus, it is likely that high ED rates are mainly driven by previous PCa treatment.

Intriguingly, the EQ-5D index value reported in the current series is comparable to those of reference populations after urethroplasty for non-radiogenic strictures [17], indicating no more, no less problems regarding the different generic health profiles. However, the mean EQ VAS score was 65, which is a patient-reported reflection of the perceived overall health state, and this was significantly lower when compared to reference mean scores of 81 [16] and 79 [17] in patients with predominantly non-radiogenic strictures. Consequently and not surprisingly, the long-winded treatment course following PCa diagnosis with the occurrence of urethral stricture as a late UAE appears to have a vital impact on the subjective health state, even though activities of daily living do not seem to be impaired.

To the best of our knowledge, this is the largest study on both objective and subjective outcomes in a cohort of patients with radiation-induced bulbomembranous stricture disease undergoing single-stage BMGU. We further evaluated comprehensive functional outcome data using a procedure-specific validated PROM tool. Our findings have direct clinical implications, particularly regarding preoperative patient counseling when harmonizing expectations with a realistic estimate.

Despite these strengths, our study has limitations. Firstly, this is a retrospective observational study and thus we were not able to include preoperative PROM data. This may hamper conclusions related to the isolated effect of BMGU on functional outcomes irrespective of previous RT. Secondly, other stricture etiologies besides the radiogenic effect could not be entirely ruled out, as we did not perform a histopathological evaluation of the stricture specimen. Given that the present work is based on a procedure-centered prospectively collected BMGU database, we were not able to provide information on patients who presented with a similar condition but underwent a different (or no) surgical procedure. Thus, a certain selection bias, which may be introduced by unmeasured patient’s and surgeon’s preference or surgical and medical limitations, cannot be ruled out.

## Conclusions

In our cohort of patients undergoing single-stage BMGU for radiation-induced urethral strictures, stricture-free survival was reasonable. However, compared to existing long-term data on non-irradiated BMGU patients, success rates and all of the functional outcome domains—LUTS, UI, and ED—are impaired. Prospective, multi-institutional collaborations should further evaluate the contributing effect of the previous PCa treatment in this context.

## Appendix

See Table 5.

**Table 5:** Frequencies and proportions of patient responses to summative questions of the EQ-5D-3L generic health profiles within the validated extension of the USS PROM tool in 38 patients who underwent 1-stage buccal mucosal graft urethroplasty for radiogenic bulbomembranous urethral stricture

EQ-5D-3L generic health profiles	Frequencies (proportions, %)
Mobility	
I have no problems in walking about	31 (82)
I have some problems in walking about	7 (18)
I am confined to bed	0 (0)
Self-care	
I have no problems with self-care	37 (97)
I have some problems washing or dressing myself	1 (2.6)
I am unable to wash or dress myself	0 (0)
Usual activities	
I have no problems with performing my usual activities	29 (76)
I have some problems with performing my usual activities	9 (24)
I am unable to perform my usual activities	0 (0)
Pain/discomfort	
I have no pain or discomfort	24 (63)
I have moderate pain or discomfort	12 (32)
I have extreme pain or discomfort	2 (5.3)
Anxiety/depression	
I am not anxious or depressed	33 (87)
I am moderately anxious or depressed	5 (13)
I am extremely anxious or depressed	0 (0)

Proportions may not add up to 100%, as they are rounded

*EQ-5D-3L* EuroQol five dimensions questionnaire, three-level version, *USS PROM* Urethral Stricture Surgery Patient-reported Outcome Measure
